# Assessment of the radiofrequency ablation dynamics of esophageal tissue with optical coherence tomography

**DOI:** 10.1117/1.JBO.22.7.076001

**Published:** 2017-07-06

**Authors:** Hsiang-Chieh Lee, Osman O. Ahsen, Jonathan J. Liu, Tsung-Han Tsai, Qin Huang, Hiroshi Mashimo, James G. Fujimoto

**Affiliations:** a Massachusetts Institute of Technology, Department of Electrical Engineering and Computer Science and Research Laboratory of Electronics, Cambridge, Massachusetts, United States; b Veterans Affairs Boston Healthcare System, Boston, Massachusetts, United States; c Harvard Medical School, Boston, Massachusetts, United States

**Keywords:** optical coherence tomography, medical and biological imaging, endoscopic imaging, gastrointestinal, ablation of tissue

## Abstract

Radiofrequency ablation (RFA) is widely used for the eradication of dysplasia and the treatment of early stage esophageal carcinoma in patients with Barrett’s esophagus (BE). However, there are several factors, such as variation of BE epithelium (EP) thickness among individual patients and varying RFA catheter-tissue contact, which may compromise RFA efficacy. We used a high-speed optical coherence tomography (OCT) system to identify and monitor changes in the esophageal tissue architecture from RFA. Two different OCT imaging/RFA application protocols were performed using an *ex vivo* swine esophagus model: (1) post-RFA volumetric OCT imaging for quantitative analysis of the coagulum formation using RFA applications with different energy settings, and (2) M-mode OCT imaging for monitoring the dynamics of tissue architectural changes in real time during RFA application. Post-RFA volumetric OCT measurements showed an increase in the coagulum thickness with respect to the increasing RFA energies. Using a subset of the specimens, OCT measurements of coagulum and coagulum + residual EP thickness were shown to agree with histology, which accounted for specimen shrinkage during histological processing. In addition, we demonstrated the feasibility of OCT for real-time visualization of the architectural changes during RFA application with different energy settings. Results suggest feasibility of using OCT for RFA treatment planning and guidance.

## 1 Introduction

Radiofrequency ablation (RFA) is being increasingly used in the treatment of various diseases including solid tumors (e.g., liver, kidney, and lung malignancies),^1^^–^^3^ arterial fibrillation (AF),^4^ and dysplasia in the esophagus.^5^ As a thermal ablation technique, RFA delivers thermal energy (heat) by applying an alternating electric current in the radiofrequency (RF) regime through electrode(s) placed either within (e.g., solid tumor) or over the targeted lesion (e.g., AF or dysplasia treatment). In current practice, most RFA procedures are performed under image guidance, such as x-ray, computed tomography, magnetic resonance imaging, ultrasound, or white light endoscopy to monitor the ablation of the targeted lesion in response to the RFA application. In addition to image guidance, methods such as temperature sensing or impedance measurement are used to provide feedback on the RFA application. However, the temporal or spatial resolution of these approaches is relatively limited; hence, it is difficult to obtain the local information on the treated lesions.

When compared with various existing modalities for treating esophageal neoplasms, such as photodynamic therapy,^6^ argon plasma coagulation,^7^ endoscopic mucosal resection (EMR),^8^ and cryospray ablation,^9^ RFA has been demonstrated as a safe and effective treatment for the complete eradication of dysplasia (CE-D) and early stage esophageal carcinoma in patients with Barrett’s esophagus (BE).^5^^,^^10^^–^^12^ Commercially available RFA devices [Barrx series, Medtronic (formerly BARRX Medical, California), Minnesota] can be generally divided into two categories based on the treatment area: (1) circumferential ablation catheters, which are introduced over a guidewire independent of the endoscope and consist of different size inflatable balloons to accommodate variations in the diameter of the esophagus among individuals (18- to 31-mm diameters with size increments of 3 mm) and (2) focal ablation catheters, which are introduced through the instrument channel or mounted on the distal end of the endoscope to target specific esophageal lesions under endoscopic guidance.^13^^,^^14^ The RF energy delivery with these devices is achieved by a thin layer bipolar electrode array that directly contacts the tissue. A leading study showed that, among patients who received RFA treatments for either nondysplastic BE [intestinal metaplasia (IM)] or any grade of the dysplasia, 78% and 91% of patients achieved complete eradication of IM (CE-IM) and CE-D, respectively.^12^ In addition, CE-IM was achieved in 92% of patients at a 5-year follow-up.^15^ Furthermore, a stricture rate of 6% was reported among RFA-treated patients, which is notably lower than alternative treatment methods for dysplasia in BE.^5^

Although RFA was effective in eradicating BE, repetitive treatment sessions were required to achieve CE-IM or CE-D. On average, 3.4 and 3.5 sessions were required for patients with IM alone^16^ or any grade of dysplasia^5^ to achieve CE-IM, respectively. Current practice requires multiple sequential RFA applications, with abrasion of the coagulum between sequential applications, to ensure sufficient ablation depth of the BE epithelium (EP).^15^ These multistep RFA procedures are recommended by the manufacturer because of the limited penetration of RF energy into superficially ablated tissue. In addition, good tissue contact with the RFA catheter is essential to achieve optimal ablation depth. Lastly, assessing the effectiveness of each RFA application mainly relies on visual inspection, which can be challenging due to bleeding from the ablated sites. Recent studies report recurrence rates of IM in 13%^5^ and 25.9%^17^ of the patients who received RFA treatment for dysplasia at 1 year after CE-IM was achieved, suggesting that the current RFA practice may not be optimal. An endoscopic imaging modality that can provide subsurface architectural information of the targeted site either before or immediately after the RFA application may be helpful for treatment planning and guidance.

Optical coherence tomography (OCT) provides cross-sectional or three-dimensional (3-D) imaging of tissue architecture in real time and is well suited for assessing RFA application. Our group previously reported the identification of structures reminiscent of subsquamous BE glands (buried BE glands) in patients before and after RFA treatment.^18^^,^^19^ This 27-patient study showed a high prevalence of buried glands in patients undergoing RFA treatment (72% of the patients before achieving CE-IM and 63% of the patients who achieved CE-IM). However, due to limited coverage of the small imaging catheter and biopsy sampling errors, a precise histologic correlation of structures identified in the *in vivo* OCT images was challenging. A study by Cobb et al.^20^ investigated the histological correlation of buried BE glands on *ex vivo* esophagectomy specimens using an ultrahigh-resolution OCT system and identified buried BE glands in 10 out of 14 specimens. However, differentiation between buried BE glands and other subsquamous glandular structures (SGSs) remained challenging for *in vivo* imaging. A more recent *in vivo* study using a commercial volumetric endoscopic OCT system with a balloon imaging catheter showed that most of the identified post-RFA SGSs corresponded to normal histologic findings rather than buried BE tissue.^21^ Furthermore, in a previous study of 33 patients undergoing RFA treatment, our group reported that the presence of residual BE glands or BE EP in the post-RFA OCT images were strong predictors for the RFA treatment response.^22^ A BE EP thickness of 333  μm or greater that was measured from the pre-RFA OCT images had a predictive value with 92.3% sensitivity and 85% specificity for the presence of BE at the follow-up visit. This suggested the potential of using OCT to guide RFA or adjust dosimetry to improve efficacy.

The use of OCT to guide RFA was first demonstrated in cardiac arrhythmia treatment. Real-time monitoring of changes in cardiac tissue architecture during RFA application was reported by using a forward-imaging catheter on the swine heart *ex vivo*^23^ and *in vivo*,^24^ demonstrating the feasibility of image-guided RFA. In addition, due to the birefringent nature of the myocardium, studies have demonstrated polarization-sensitive OCT (PS-OCT) imaging with a fiber-optic forward-imaging catheter for cardiac RFA monitoring.^25^ A later study reported that an RFA catheter enabled concurrent RFA application and polarization-sensitive low-coherence interferometry measurement on the swine heart *ex vivo*.^26^ However, a detailed investigation using OCT to identify and monitor the changes in esophageal tissue architecture during RFA application has not been performed.

In this study, we used a high-speed OCT system to identify and monitor changes in *ex vivo* swine esophageal tissue by using commercially available RFA catheters. Two different OCT imaging/RFA application protocols were performed: (1) post-RFA volumetric OCT imaging to measure the coagulum formation produced by RFA applications with different energies (dosages), and (2) M-mode OCT imaging to monitor the dynamic changes in tissue architecture during the RFA application. In addition, we obtained histology on a subset of specimens to investigate the relationship between OCT measurements of coagulum and coagulum + residual EP thickness when compared with histology.

## 2 Materials and Methods

### 2.1 High-Speed Swept-Source Optical Coherence Tomography System

**Fig. 1:**
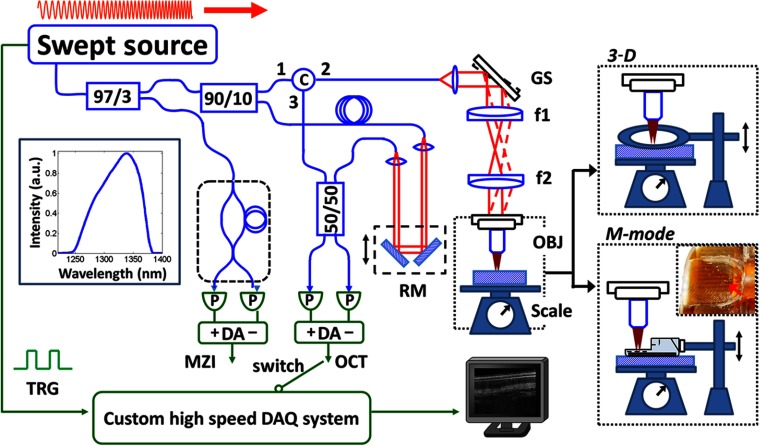
Schematic diagram of the high-speed swept-source OCT system (blue: optics; green: electronics). Inset (left): swept light source spectrum showing a near Gaussian spectral shape. Inset (top right): imaging setup showing volumetric (3-D) OCT imaging, where a cover glass was pressed over the specimen with a controlled pressure measured by the scale below. Inset (lower right): imaging setup for M-mode OCT, where the beam was scanned through the ablation device. MZI: Mach–Zehnder interferometer; TRG: trigger signal; DA: differential amplifier; RM: reference mirror; GS: galvanometer scanner; C: circulator; OBJ: objective; f1, f2: scan lens and tube lens of the relay optics; and DAQ: data acquisition.

Figure 1 shows the schematic diagram of the high-speed swept-source OCT system used in this study. The system employed a double-buffered Fourier domain mode-locked (FDML) laser that enabled an effective A-scan rate of ∼240  kHz with an output optical power of ∼60  mW, a sweep range of ∼140  nm, and a bandwidth of ∼90  nm full width at half maximum (FWHM) centered at 1.3-μm wavelength. The configuration of the FDML light source was similar to previously reported studies.^27^^,^^28^

Light emitted from the FDML laser was split and coupled into a Mach–Zehnder interferometer (MZI) and an OCT interferometer. The interference fringes from the MZI were detected by a dual-balanced detector (PDB430C, Thorlabs, Inc., New Jersey) with a 350-MHz detection bandwidth and were acquired once before the OCT imaging session to recalibrate the OCT interference signals. The OCT interferometer consisted of a 90/10 fiber-optic coupler, an optical circulator (AC Photonics, California), a sample arm with a scanning microscope,^29^ and a single-pass reference arm. In the microscope, a pair of closely spaced galvanometer scanners with 5-mm mirrors (6215 H, Cambridge Technology, Inc., Massachusetts) was used to provide two-dimensional (2-D) beam scanning of the specimen. Light from the FDML was collimated onto the scanner by using a near-infrared (NIR) achromatic lens (f=18  mm) and then relayed to the objective by two NIR achromatic doublets (f1=f2=75  mm). A long working distance NIR objective (WD=37.5  mm, M Plan Apo NIR 5×, Mitutoyo, Japan) was used to provide sufficient space for the RFA catheter and tissue mounting as well as to achieve high transverse image resolution.

Light from the sample and reference arms was interfered in the 50/50 fiber-optic coupler (AC Photonics, California) and detected by using a dual-balanced detector with identical specifications to the MZI detector. Both the calibration (MZI) and OCT signals were digitized by using an A/D card (ATS9350, Alazar Tech, Canada) at 500  MS/s. The measured system sensitivity was 107 dB with an incident power of ∼24  mW onto the sample, and the 6-dB sensitivity roll-off depth was ∼2.5  mm (in air). The axial image resolution was 7.4  μm (FWHM in tissue, assuming a tissue refractive index of 1.38 at ∼1310-nm wavelength), and a transverse resolution of 12.1  μm (FWHM in air) was characterized by using the knife-edge method. The relatively fine transverse resolution yielded a short depth of focus (DOF) of ∼500  μm.

### 2.2 Radiofrequency Ablation Setup

A commercial RFA instrument with a focal RFA catheter (Barrx Flex, Model 90-9000 and Barrx 90, Medtronic, Minneapolis) was used in this study. The instrument allowed adjustment of the RF energy settings between 12 and $15\;J/{\mathrm{cm}}^{2}$ (power density: $40\;W/{\mathrm{cm}}^{2}$, frequency: 460 kHz, duration: ∼0.3  s) for the Barrx 90 catheter. The catheter ablation paddle was $13\times 20\;{\mathrm{mm}}^{2}$ and covered by an electrode array of 24 electrodes. Each electrode was 250-μm wide with a separation of 250  μm from the adjacent electrodes. To improve light transmission and reduce aberration through the ablation paddle, a small region ($∼4\times 4\;{\mathrm{mm}}^{2}$) on the back of the paddle was machined to thin supporting plastic material without affecting the electrodes' integrity. The modified ablation paddle is shown in the lower right inset in Fig. 1, where the region (red arrow) allows OCT imaging during RFA application.

### 2.3 Specimen Handling, Optical Coherence Tomography Imaging, and Radiofrequency Ablation Protocol

Fresh swine esophagi were excised and stored in Dulbecco’s Modified Eagle’s Media (Cellgro, Corning, Virginia) at 4°C. Before the RFA application, the swine esophagus was dissected from the distal end (stomach side) to proximal end (larynx side) into $∼2\times 3\;{\mathrm{cm}}^{2}$ specimens to ensure sufficient contact between the RFA catheter (ablation paddle) and specimen. RFA was performed with specimens at room temperature, and each specimen was then sequentially ablated with different RFA energy dosages in individual protocols in order to avoid biased measurements caused by tissue variations between individual animals. In this study, two OCT imaging protocols with different RFA settings were performed in order to investigate the coagulum formation due to RFA application. A total of 24 specimens were collected from six swine esophagi [four specimens per esophagus (mean, range: 3 to 6)]. Previous studies on *ex vivo* human colon tissues reported a decrease in the EP thickness measured in the OCT images with increasing pressure exerted by the imaging catheters.^30^ Therefore, during the OCT imaging, controlled pressure was applied in order to reduce the variability of the thickness measurements. The force exerted by an endoscope to a distally mounted focal ablation catheter was measured as ∼100  g over the same surface area as the ablation paddle ($∼1.3\times 2\;{\mathrm{cm}}^{2}$), corresponding to a pressure of $∼38\;g/{\mathrm{cm}}^{2}$. Details of the individual protocols are described below.

1. Post-RFA volumetric OCT imaging: This protocol investigated the feasibility of using OCT to quantitatively assess the coagulum produced by RFA applications with different energy settings. In this protocol, a single RFA application was performed on multiple *ex vivo* swine esophagus specimens using different energies: $12\;J/{\mathrm{cm}}^{2}$ (N=4), $13\;J/{\mathrm{cm}}^{2}$ (N=3), $14\;J/{\mathrm{cm}}^{2}$ (N=2), and $15\;J/{\mathrm{cm}}^{2}$ (N=4). The range of energy settings was limited to 12 to $15\;J/{\mathrm{cm}}^{2}$ by the commercial RFA instrument. A thin cover glass ($2.4\times 3\;{\mathrm{cm}}^{2}$) was pressed over the ablated specimen (top-right inset, Fig. 1) to create a flat imaging plane and maintain a constant pressure ($∼220\;g/6\;{\mathrm{cm}}^{2}$), consistent with the pressure exerted by the RFA paddle on the tissue ($100\;g/2.6\;{\mathrm{cm}}^{2}$). In this study, the imaging field was limited by the 5× objective. Since the coagulum was nonuniform across the ablated area ($∼2\times 3\;{\mathrm{cm}}^{2}$), post-RFA volumetric OCT imaging was performed on an area of $3\times 3\;{\mathrm{mm}}^{2}$ (1000×1000 A-scans) sampling the ablated region with the most prominent coagulation.
2. M-mode OCT imaging: This protocol investigated the feasibility of using OCT for the real-time monitoring of tissue architecture changes during the RFA application. In this protocol, a modified focal RFA catheter was placed over the specimens with a controlled pressure ($100\;g/2.6\;{\mathrm{cm}}^{2}$) as described in protocol (1). Repetitive B-scan OCT images (M-mode OCT obtained through the spaces between electrodes on the RFA catheter) were acquired during the RFA application (lower right inset, Fig. 1) at 240 frames per second. Each frame consisted of 1000 A-scans. Unlike the first protocol, two consecutive RFA applications with the energy settings of either $12\;J/{\mathrm{cm}}^{2}$ or $15\;J/{\mathrm{cm}}^{2}$ were performed with M-mode OCT imaging during both applications. Furthermore, the coagulum resulting from the first RFA application was not removed before the second RFA application in order to investigate the ablation efficacy of the second RFA application in the presence of existing coagulum. In current clinical practice using a focal Barrx RFA catheter, a region is treated with two RFA applications without removing the RFA paddle between these applications; then, the coagulum is removed by abrading with the catheter, and two additional RFA applications are performed. Our study [protocol (2)] mimicked the first part of the clinical RFA protocol and investigated the first two RFA applications.

### 2.4 Quantitative Analysis of Optical Coherence Tomography and Histology Data

Following the RFA application and OCT imaging, the specimens used in both protocols were inked to indicate the OCT imaged area, placed in standard tissue cassettes, and fixed in 10% formalin prior to histology processing. Standard hematoxylin and eosin (H&E) staining was used to assess the coagulum from the RFA application.^13^^,^^14^ Digital pathology images were captured from the H&E histology slides by using a slide scanner (Aperio AT2, Leica Biosystems, Illinois) with a 20× magnification. Both the OCT images and corresponding H&E histology images were analyzed to identify the coagulum characteristics, including the coagulum thickness.

Post-RFA OCT images were examined in order to segment the coagulum, residual EP, and underlying lamina propria (LP) layers manually, based on the difference in scattering properties. The thickness of the coagulum and coagulum + residual EP layer was measured in individual cross-sectional OCT images by assuming a tissue refractive index of 1.38 at ∼1310-nm wavelength.^31^ A 2-D thickness map was generated from volumetric post-RFA OCT datasets. The average, as well as minimum and maximum thickness, was obtained from the OCT measurements of individual specimens (volumetric or single cross-sectional OCT images) in order to assess the effects of RFA. For the subset of specimens processed for histology, ∼4 to 5 histological sections with a step interval of 200  μm were obtained from the OCT imaged region. The thickness of the coagulum and residual EP layer was manually measured at multiple locations using a digital pathology viewer (Aperio ImageScope v.11, Leica Biosystems, Illinois). Similar to the OCT measurement, the average, as well as minimum and maximum thickness, was measured and compared with the corresponding OCT measurement.

## 3 Results

### 3.1 Radiofrequency Ablation Coagulum Thickness Analysis

**Fig. 2:**
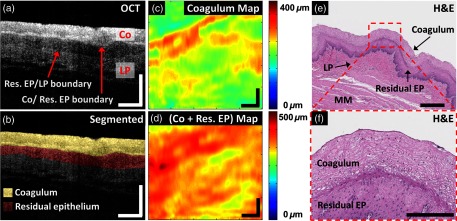
Coagulated tissue analysis. (a) Cross-sectional OCT image from an RFA-treated *ex vivo* swine esophagus specimen shows tissue architecture reminiscent of the coagulum (Co), the residual EP (Res. EP), and underlying LP layer. (b) Cross-sectional OCT image of (a) with the coagulum and residual EP layers manually segmented to generate 2-D thickness maps of the (c) coagulum and (d) coagulum + residual EP layer, respectively. (e) Representative H&E stained histology confirms the three-layer architecture observed in (a). (f) The zoomed-in histology image over the region of interest (red dashed box) marked in (e) shows characteristic features of the coagulum. Scale bars: (a–e): 500  μm; (f): 200  μm.

Figure 2 shows the cross-sectional OCT and representative H&E histology images from a swine esophagus specimen treated with a single RFA application of $14\;J/{\mathrm{cm}}^{2}$. As shown in Fig. 2(a), a three-layer tissue architecture, a homogenous hyperscattering layer near the tissue surface corresponding to the coagulum (Co), a thin hyposcattering layer corresponding to the residual EP layer, and a hyperscattering layer corresponding to the LP layer were identified and manually segmented [Fig. 2(b)]. 2-D thickness maps of the coagulum and coagulum + residual EP layer [Figs. 2(c) and 2(d)] from the segmented volumetric OCT datasets are also shown. Varying thickness of the coagulum and coagulum + residual EP layer can be observed across the imaging field, thus suggesting nonuniformity of the coagulum. The comparison of the coagulum and coagulum + residual EP layer thickness between OCT and histology is presented in the subsequent section. This three-layer tissue architecture was consistent with the corresponding histology image [Fig. 2(e)]. In the zoomed-in histology image [Fig. 2(f)], the coagulum is visible as layers of squamous cells with intracellular voids, which may cause the associated hyperscattering of the coagulum in the OCT images.

### 3.2 Volumetric Optical Coherence Tomography Imaging of the Radiofrequency Ablation Coagulum for Different Radiofrequency Energy Settings

**Fig. 3:**
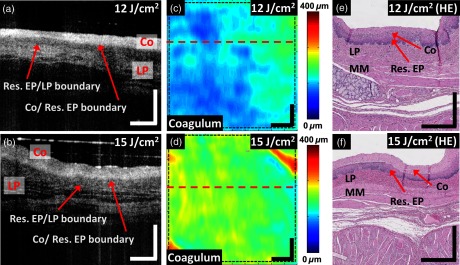
Coagulum thickness analysis from specimens ablated with single RFA applications at different RF energies (12 and $15\;J/{\mathrm{cm}}^{2}$). (a, b) Cross-sectional OCT images corresponding to a location indicated in the (c, d) coagulum thickness maps. (e, f) H&E stained histology images close to the locations of the respective (a, b) cross-sectional OCT images. Scale bars: (a–f): 500  μm. Res. EP: residual epithelium; Co: coagulum; LP: lamina propria; MM: muscularis mucosa.

The aim of protocol (1) was to investigate the feasibility of using OCT to identify and measure the changes of coagulum thickness in individual specimens after single RFA applications with different RF energy settings. Figure 3 shows the (a, b) cross-sectional OCT images as well as (c, d) thickness maps of the coagulum layer from two representative specimens ablated with 12 and $15\;J/{\mathrm{cm}}^{2}$, respectively. The fraction of the residual EP layer in the specimen treated with a higher energy RFA ($15\;J/{\mathrm{cm}}^{2}$) was decreased when compared with one treated with a lower energy RFA ($12\;J/{\mathrm{cm}}^{2}$). The coagulum thickness for different energy RFA applications can also be seen in the 2-D coagulum thickness maps [Figs. 3(c) and 3(d)]. Figures 3(e) and 3(f) show histology from the locations near the cross-sectional OCT images [Figs. 3(a) and 3(b)], where the boundary between the coagulum and residual EP layer was not as discernable in the specimen treated with $12\;J/{\mathrm{cm}}^{2}$ [Fig. 3(e)] when compared with $15\;J/{\mathrm{cm}}^{2}$ [Fig. 3(f)].

**Fig. 4:**
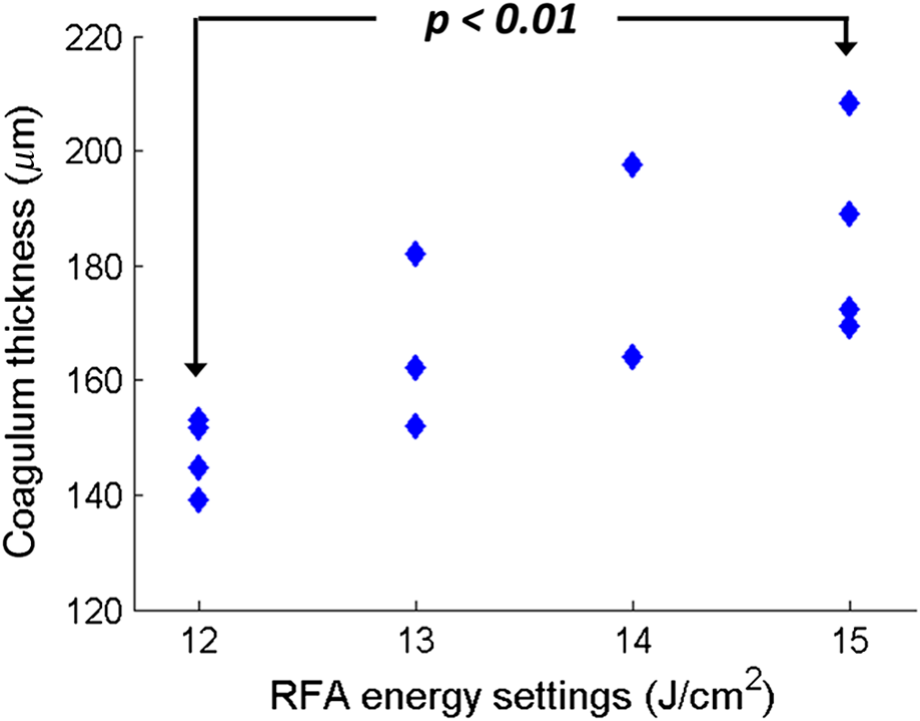
Coagulum thickness measured in the post-RFA volumetric OCT datasets of individual *ex vivo* swine esophagus specimens treated with single RFA applications at different RF energy settings (12 to $15\;J/{\mathrm{cm}}^{2}$). The average coagulum thickness measured in the volumetric OCT datasets of individual specimens was used to represent the coagulum thickness due to RFA with the designated energies.

The average coagulum thickness in individual specimens was measured by using the segmented post-RFA volumetric OCT datasets. The coagulum thickness increased with increasing ablation energy (Fig. 4), as expected. A statistically significant difference (p<0.01, student t-test) was observed in the coagulum thickness at 15 versus $12\;J/{\mathrm{cm}}^{2}$. Table 1 shows the thickness of the coagulum and coagulum + residual EP layer measured in the OCT images versus the corresponding H&E histology images by using a subset of specimens ($12\;J/{\mathrm{cm}}^{2}$: N=2; $15\;J/{\mathrm{cm}}^{2}$: N=2), including the two specimens shown in Fig. 3. A variation of the average thickness of the coagulum + residual EP layer, which may be attributed to the thickness variation of the EP layer prior to RFA application, was observed in the OCT measurements, because the specimens were collected from different swine and different longitudinal locations from the esophagi. In histology, one of the specimens treated with a higher energy RFA (specimen C) showed comparable coagulum thickness to one treated with a lower energy (specimen A). This may be related to specimen handling and fixation. Specimens shrink during fixation, which makes the comparison of the absolute thickness measurement between the OCT and histology images difficult. To account for shrinkage, we computed the ratio of the OCT measurement to the histology measurement for the coagulum or coagulum + residual EP layer thickness in order to compare OCT with histology. Table 1 shows that this ratio (OCT/H&E) for the coagulum is similar to the coagulum + residual EP layer among the four specimens evaluated. This suggests that, although shrinkage varied between specimens, it was consistent among different tissue types (e.g., the coagulum versus the residual EP layer).

**Table 1:** Comparison of the coagulum versus coagulum + residual EP thickness measurements between the OCT and H&E histology images.

	Coagulum thickness^a^			Coagulum + residual EP thickness^a^		
	OCT (μm)	H&E (μm)	OCT/H&E	OCT (μm)	H&E (μm)	OCT/H&E
Specimen A ($12\;J/{\mathrm{cm}}^{2}$)	140.3 (20.5)	117.7 (16.4)	1.19	220.8 (53.4)	176.7 (36.4)	1.25
Specimen B ($12\;J/{\mathrm{cm}}^{2}$)	147.8 (20)	144.1 (28.2)	1.03	251 (35.6)	264.4 (51.5)	0.95
Specimen C ($15\;J/{\mathrm{cm}}^{2}$)	207.3 (26.9)	117.5 (18.8)	1.76	362.3 (58.7)	219.1 (41.5)	1.65
Specimen D ($15\;J/{\mathrm{cm}}^{2}$)	184.7 (16.8)	177.8 (26.8)	1.04	202.9 (22.7)	206.7 (36.2)	0.98

a mean (standard deviation).

### 3.3 Concurrent Optical Coherence Tomography Imaging of Radiofrequency Ablation

**Fig. 5:**
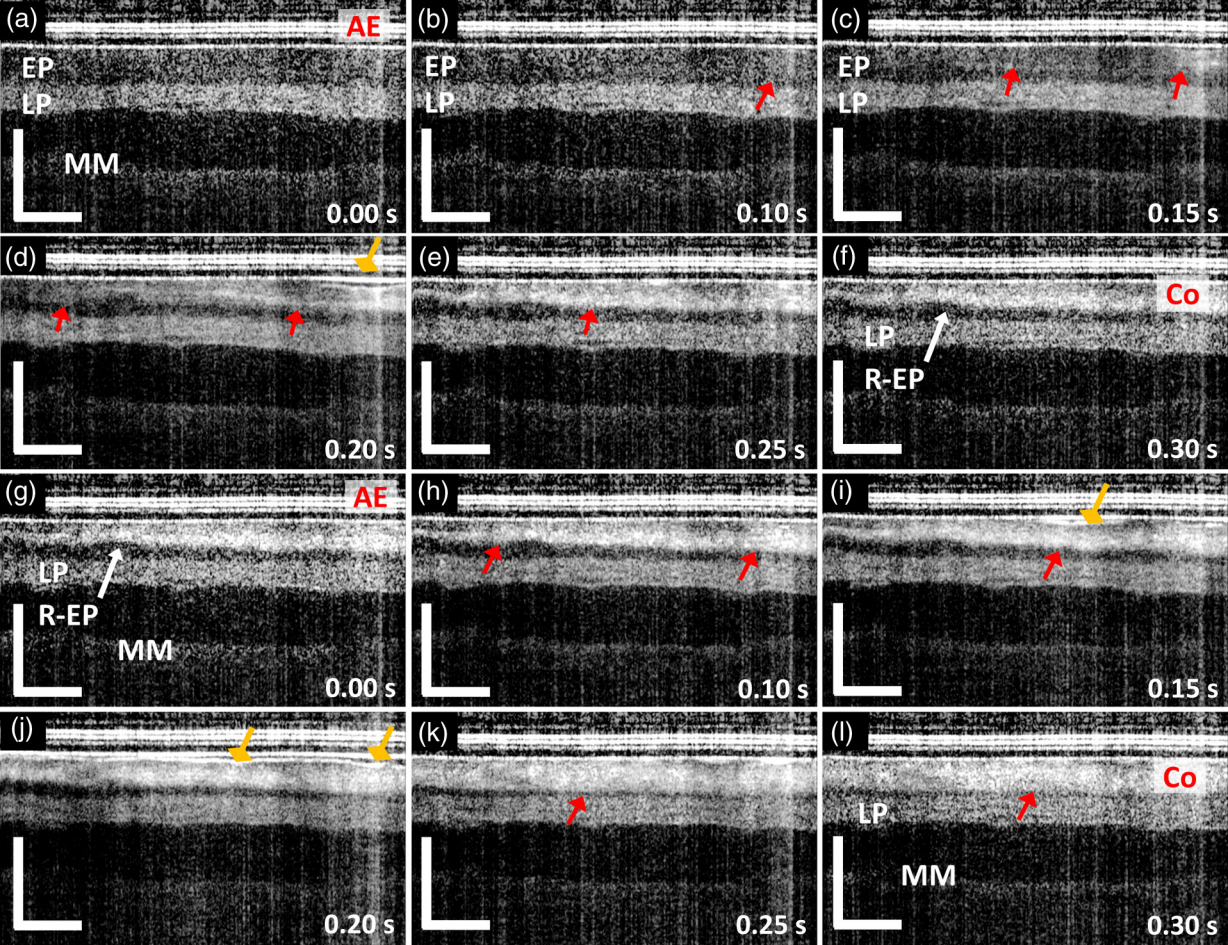
Dynamics of RFA with an energy density of $12\;J/{\mathrm{cm}}^{2}$ during the first (visualization 1) and second RFA applications (visualization 2). (a) The distinctive layered structure of swine esophagus, including the EP, LP, MM, and the RFA catheter AE layer can be clearly observed at time T=0.00  s. (b–e) Changes in the tissue architecture due to the RFA application include generation of a hyperscattering layer (red arrows) and changes in the specimen position and tissue contact (diamond arrows) starting around time T=0.10  s to 0.30 s. (f) At time T=0.30  s, the changes in tissue architecture stopped after the conclusion of the RFA energy deposition (duration: ∼0.3  s), and residual nonablated EP layer (R-EP) can be observed. (g–k) During the second RFA application, the tissue architectural changes were similar to those in (a–e). (l) At time T=0.30  s, the tissue architectural changes stopped, as in the first RFA application, and the boundary of the hyperscattering layer overlapped with the initial EP/LP boundary in most of the OCT imaging field (red arrow). s: second. Scale bars: 500  μm.

Protocol (2) was designed to demonstrate the use of OCT for real-time monitoring of tissue changes during RFA. Commercially available focal RFA catheters were modified, as described in Sec. 2.3, to enable concurrent OCT imaging during the RFA application. Two consecutive RFA applications were applied to individual specimens with energy settings of $12\;J/{\mathrm{cm}}^{2}$ (N=5) or $15\;J/{\mathrm{cm}}^{2}$ (N=6). Figure 5 shows the representative dynamics of tissue architectural changes during the first (a–f) and second (g–l) RFA application at $12\;J/{\mathrm{cm}}^{2}$. During the first RFA application, at time T=0.00  s, regular esophageal structures [EP, LP, and part of the muscularis mucosa (MM)] were observed beneath the RFA ablation electrodes (AE). At time T=0.10 to 0.25 s, hyperscattering regions corresponding to the coagulum were created by the thermal energy delivery. This also caused water vaporization over or within the specimen and, subsequently, the change in the position of the AE layer and the underlying specimen [diamond arrows, Fig. 5(d)]. This was due to water vapor ventilation, as noticed in the M-mode OCT images (visualization 1). Blurring of the tissue speckle pattern was also related to the tissue motion induced by heating and water vaporization [Figs. 5(c)–5(e)]. After T=0.30  s [Fig. 5(f)], after RFA energy deposition concluded (duration: ∼0.3  s), the boundary of the hyperscattering layer becomes stationary (arrows) and is delineated clearly, thereby allowing measurement of coagulum thickness, similar to Sec. 3.2. Below the coagulum, a thin hyposcattering layer corresponding to the residual EP layer is observed [Fig. 5(f)]. The second RFA application was performed without removing the coagulum [Figs. 5(g)–5(l)]. At time T=0.10 to 0.25 s, there was a slight loss of electrode-tissue contact and blurring of the tissue speckle pattern, similar to the first RFA [Figs. 5(i)–5(k)]. The depth of the hyperscattering layer further increased and became more uniformly distributed after the second RFA application. In addition, the boundary of the hyperscattering layer (coagulum) nearly overlapped with the initial EP/LP boundary in two-thirds of the imaging field, thus suggesting the EP layer was fully ablated over these regions. However, distinct architectural changes in the LP layer could not be identified in the OCT image after the second RFA application, thereby making it difficult to assess the depth of the ablation.

**Fig. 6:**
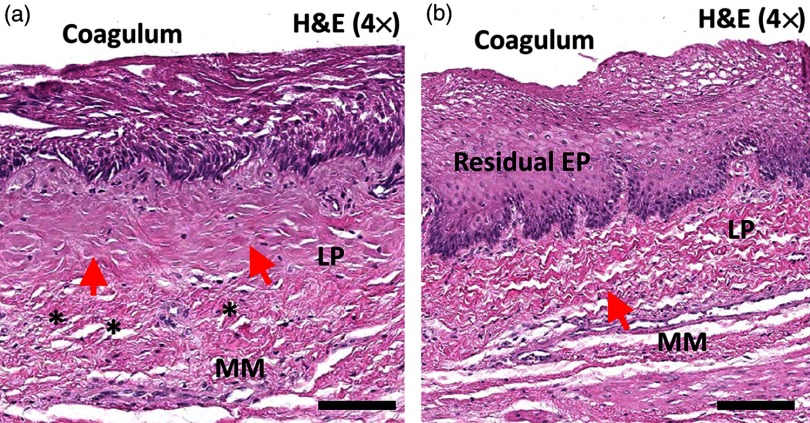
Representative H&E stained histology images (a) near the imaged site as Fig. 5 and (b) from a location ∼5  mm away from (a). (a, b) Layers of squamous cells with intracellular spaces corresponding to the RFA coagulum, after two RFA applications, can be identified. Distinctive architectural appearance of the superficial part of the LP layer (red arrows) was observed in (a) when compared with the deeper part of the LP layer (stars, a) and (b) the LP layer from a different location (red arrows, b), thus suggesting the thermal energy delivery extended into the LP layer in (a). Scale bars: 200  μm.

Figure 6 shows the H&E histology image (a) near the OCT imaged site of Fig. 5 and (b) from a location ∼5  mm away from (a), but still within the ablated region. Similar to Fig. 2(b), the coagulum was characterized by layers of squamous cells with intracellular voids. In addition, the superficial LP layer near the OCT imaged site exhibited a different architectural appearance [arrows, Fig. 6(a)] when compared with the LP layer away from the OCT imaged site [arrows, Fig. 6(b)]. This difference can also be identified by comparing the tissue architecture of the superficial part of the LP layer (arrows) and the deeper part of the LP layer (stars) in Fig. 6(a). Both observations confirmed that the ablation extended from the EP into the superficial LP layer over the OCT imaged site, where the coagulum boundary nearly overlapped with the initial EP/LP boundary in most of the OCT imaging field. While both histology images were acquired within the regions treated with two consecutive RFA applications, the difference in the appearance of the superficial LP layer suggests that the thermal energy delivery was nonuniform across the ablated regions. The coagulum thickness observed in Fig. 6(a) was thinner when compared with Fig. 6(b), which may be related to sloughing of the coagulum during removal of the ablation catheter or the fixation process.

**Fig. 7:**
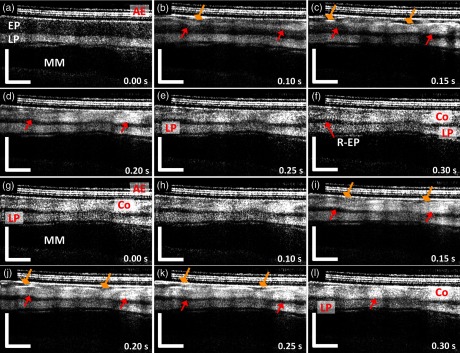
Dynamics of RFA with an energy density of $15\;J/{\mathrm{cm}}^{2}$ during the first (visualization 3) and second RFA applications (visualization 4). (a) The distinctive layered structure of swine esophagus, including the EP, LP, MM, and RFA catheter AE layer can be clearly differentiated. (b–e) Changes in the tissue architectures due to RFA, including the generation of a hyperscattering layer (red arrows) and changes in the specimen position and tissue contact (diamond arrows), were observed at time T=0.10  s to 0.30 s. (f) At time T=0.30  s, the changes in tissue architecture stopped after RFA deposition concluded (duration: ∼0.3  s), and only a limited fraction of residual nonablated EP (R-EP) was observed due to the higher RF energy. (g–k) The architectural changes during the second application were similar to (a–e). (l) At time T=0.30  s, the changes stopped, and the boundary of the hyperscattering layer overlapped the initial EP/LP boundary in most of the OCT imaging field (red arrow). s: second. Scale bars: 500  μm.

Figure 7 shows the dynamics of tissue architecture changes from a specimen treated with two consecutive RFA applications of $15\;J/{\mathrm{cm}}^{2}$. The changes observed during the (a–f) first and (g–l) second RFA applications were similar to those in Fig. 5. However, due to the higher energy, the hyperscattering layer corresponding to the coagulum was thicker after the first RFA application. The boundary of the coagulum nearly overlapped the initial EP/LP boundary in some portions of the OCT imaging field [red arrow, Fig. 7(f)], thus suggesting a complete EP ablation over these regions. After the second RFA application [Figs. 7(g)–7(l)], the boundary of the hyperscattering layer overlapped the initial EP/LP boundary in nearly the entire OCT imaging field. In addition, no significant architectural changes within the LP layer as a result of the RFA application could be identified in the OCT image even though a higher energy setting was used. This makes it difficult to measure the increase in coagulum thickness resulting from the second RFA application.

Table 2 summarizes the coagulum thickness measured in the M-mode OCT images of individual specimens after the first RFA application. Similar to Fig. 4, the specimens treated with $15\;J/{\mathrm{cm}}^{2}$ RFA applications showed thicker coagulum than those treated with $12\;J/{\mathrm{cm}}^{2}$. In addition, although the coagulum thickness after the first RFA application listed in Table 2 is higher than those reported in the previous section (Fig. 4 and Table 1), it should be noted that only cross-sectional OCT images from a single location were used in the analysis, rather than a volumetric OCT dataset as in first imaging protocol. Lastly, as shown in Figs. 5 and 7, there were no discernible OCT changes in the tissue scattering properties within the LP layer resulting from the RFA applications. Therefore, using OCT to measure the increase in the coagulum thickness from the second RFA application after it reached the depth of the LP layer was difficult.

**Table 2:** Coagulum thickness from two different energy settings measured in M-mode OCT after the first RFA application.

	$12\;J/{\mathrm{cm}}^{2}$ (N=5)	$15\;J/{\mathrm{cm}}^{2}$ (N=6)	p-value
Maximum coagulum thickness (μm), mean (standard deviation).	195.2 (17.9)	229.4 (17.8)	0.012
Average coagulum thickness (μm), mean (standard deviation)	168.3 (15.3)	201.5 (21.2)	0.015
Minimum coagulum thickness (μm), mean (standard deviation)	122.6 (19.5)	167.8 (27.8)	0.012

## 4 Discussion

In this study, we demonstrated the feasibility of using OCT to measure the coagulum thickness after different energy RFA applications with an *ex vivo* swine esophagus model. In protocol (1), post-RFA volumetric OCT images showed an increase in the coagulum thickness with increasing RFA energies, thus suggesting the feasibility of controlling coagulum thickness (i.e., the ablation depth) by adjusting RFA energy settings. In protocol (2), real-time OCT imaging and RFA demonstrated *in situ* measurement/assessment of the coagulum and residual EP during different energy RFA applications. This measurement used repeated M-mode cross-sectional OCT images from a fixed position on each specimen, which may not correspond to the region with the deepest coagulum formation. Nevertheless, findings similar to those reported with post-RFA volumetric OCT in protocol (1) were observed. The results of protocols (1) and (2) suggest the potential to control the ablation depth by adjusting RFA energies and using OCT to monitor thermally induced changes in tissue architecture in real time, as well as to assess ablation depth after RFA.

There were several limitations in this study. First, OCT imaging was performed with a 5× objective, which achieved high resolution of architectural features but limited the imaging field and DOF. Due to the varying contact between the esophagus specimen and the RFA catheter, the thermal energy delivery (i.e., the coagulum formation) may not be uniform over the entire ablated region. Given the limited imaging field and the nonuniform coagulum formation, in protocol (1), post-RFA volumetric OCT imaging was performed over regions exhibiting the most prominent coagulum formation, rather than the entire ablated region. Therefore, the measurements may underestimate the variation of RFA ablation depths.

In addition, as shown in Table 1, there was disagreement between the OCT and histology measurements of the coagulum and the coagulum + residual EP layer thicknesses. Different specimens exhibited different degrees of shrinkage during histological processing. This varying tissue shrinkage was related to the specimens not being fixed in the same imaging mount as the one used during the OCT imaging or RFA application, where a controlled pressure was applied. Because an esophagus is highly elastic, specimen handling and not having fixed the specimen with the same pressure may have led to a variation of tissue thickness along the transverse direction. Therefore, the scale factor between OCT and histology varied between specimens, although the ratio of coagulum and coagulum + residual EP layer was relatively consistent. In addition, part of the RFA coagulum may have sloughed off during the tissue preservation process and affected the histology measurements.

Furthermore, the thickness of the coagulum and residual EP layer measured in the histology images may be vulnerable to sampling error. For this reason, we collected ∼4 to 5 serial histology sections at intervals of 200  μm from the OCT imaged area, where the specimen was inked to facilitate the coregistration of the OCT image with histology. Also, in each histology section, the thickness measurement was performed at multiple locations near or within the OCT imaged area to avoid biasing the measurement toward certain extreme values. However, we cannot completely rule out the presence of sampling error, which may have contributed to the disagreement.

Future studies can mitigate the thickness variation by designing a specimen holder, which enables RFA application and OCT imaging with a controlled pressure on the specimen, as well as subsequently fixing the specimens in the same specimen holder to reduce distortion from tissue handling and tissue shrinkage.^32^^,^^33^ Baseline OCT measurements of the unablated specimen can be used to control tissue shrinkage. These modifications, in combination with performing OCT imaging with a larger field of view, may improve the histological correspondence and reduce potential sampling errors.

As a preliminary feasibility study, the main focus of protocol (2) was to visualize the ablation depth, the time dependence of the ablation zone, and the efficiency of the RFA application with or without the presence of coagulum. Therefore, the exact clinical treatment protocol was not followed, in which the coagulum is removed by mechanically abrading it after two RFA applications and two additional RFA applications are performed. It is challenging to perform the abrasion exactly across different specimens, and we believe this may introduce additional confounding factors to the study findings. Moreover, adding the abrasion and investigating the effects of two additional RFA applications would increase the number of specimens required for histology.

Using OCT to identify the architectural changes within the LP layer due to the RFA applications was difficult (Figs. 5 and 7). The LP is a thin layer mixed with connective tissue and glandular tissues/microvascular networks. However, the LP layer also has collagen, which exhibits strong birefringence in connective tissue, and the contrast of the LP layer can be enhanced using PS-OCT.^34^^–^^36^ Therefore, it may be possible to differentiate ablated from nonablated LP by using the loss of birefringence from thermally induced collagen denaturation.^37^

This study uses H&E to assess tissue architectural changes from RFA, similar to previous studies.^13^^,^^14^^,^^38^ Although H&E staining can identify immediate physical tissue damage from coagulation, it may not identify subsequent changes in cell viability from thermal injury, i.e., apoptosis/necrosis, which are of biological importance in an *in vivo* situation. Thus, future studies incorporating the cell viability stains, such as nitroblue tetrazolum chloride (NBTC) histology,^32^^,^^33^ in parallel with conventional H&E staining, should be considered. However, even for *in vivo* studies, viability stains may not account for later changes in tissue destruction, such as from immune cells and delayed apoptosis after the RFA application.

Lastly, an *ex vivo* normal swine esophagus model was used in this study. The tissue ablation of the squamous mucosa in *ex vivo* swine esophagus will differ from an *in vivo* swine esophagus, which exhibits blood circulation and postablation bleeding. Studies of RFA for the treatment of liver tumors show the thermal lesion size decreases as the lesion blood perfusion rate increases,^39^ thus suggesting the importance of blood flow, which is absent in the *ex vivo* swine esophagus model. Therefore, in the future, it is important to perform *in vivo* studies to confirm these findings, since these physiological factors will likely affect the RFA dynamics. In addition, bleeding from the RFA-treated site may affect the quality of the OCT images during or post-RFA application. Although excess blood on the tissue surface can be flushed away with water irrigation, the potential degradation of OCT image quality due to bleeding requires further investigation with *in vivo* studies. Finally, normal squamous EP differs from human BE mucosa. However, there are no suitable large animal models of BE. Future studies using *ex vivo* human EMR or esophagectomy specimens may be warranted; however, these specimens are often required for diagnosis and, therefore, they are difficult to obtain. Furthermore, both EMR and esophagectomy specimens would be *ex vivo.*

## 5 Conclusion

In this study, we applied high-speed OCT to identify and monitor the changes in the esophageal tissue architecture from RFA by using commercially available clinical RFA catheters. An investigation of the coagulum thickness from different energy RFA was performed during and after individual RFA applications by using *ex vivo* swine esophagus specimens. We demonstrated the use of OCT to identify and measure the increase of the coagulum thickness with increasing RFA energy and to visualize these changes rapidly in real time. These results suggest that OCT can be integrated with RFA catheters for pretreatment exposure planning, real-time control during RFA, and posttreatment assessment to measure ablation depths and uniformity. These improvements may ultimately reduce the number of RFA treatment sessions required for CE-IM/CE-D and may also improve treatment outcomes.
